# Evaluation of a support worker role, within a nurse delegation and supervision model, for provision of medicines support for older people living at home: the Workforce Innovation for Safe and Effective (WISE) Medicines Care study

**DOI:** 10.1186/s12913-015-1120-9

**Published:** 2015-10-06

**Authors:** Cik Yin Lee, Christine Beanland, Dianne Goeman, Ann Johnson, Juliet Thorn, Susan Koch, Rohan A. Elliott

**Affiliations:** Royal District Nursing Service, RDNS Institute, 31 Alma Road, St Kilda, Victoria 3182 Australia; Monash University Centre for Medicine Use and Safety, 381 Royal Parade, Parkville, Victoria 3052 Australia; Monash University, Central Clinical School, Faculty of Medicine, Nursing and Health Sciences, Commercial Rd, Prahran, Victoria 3004 Australia; Royal District Nursing Service (Koonung), 690 Elgar Road, Box Hill, Victoria 3129 Australia; Austin Health Aged Care Services, P.O. Box 5444, Heidelberg West, Victoria 3081 Australia; Austin Health, Pharmacy Department, P.O. Box 5444, Heidelberg West, Victoria 3081 Australia

**Keywords:** Medicines support, Medication management, Older people, Elderly, Support workers, Nursing assistants, Community care workers, Community nurses

## Abstract

**Background:**

Support with managing medicines at home is a common reason for older people to receive community nursing services. With population ageing and projected nurse shortages, reliance on nurses may not be sustainable. We developed and tested a new workforce model: ‘Workforce Innovation for Safe and Effective (WISE) Medicines Care’, which enabled nurses to delegate medicines support home visits for low-risk clients to support workers (known as community care aides [CCAs]). Primary study aims were to assess whether the model increased the number of medicines support home visits conducted by CCAs, explore nurses’, CCAs’ and consumers’ experiences with the CCAs’ expanded role, and identify enablers and barriers to delegation of medicines support.

**Methods:**

A prospective before-after mixed-methods study was conducted within a community nursing service that employed a small number of CCAs. The CCAs’ main role prior to the WISE Medicines Care model was personal care, with a very limited role in medicines support. CCAs received training in medicines support, and nurses received training in assessment, delegation and supervision. Home visit data over two three-month periods were compared. Focus groups and interviews were conducted with purposive samples of nurses (*n* = 27), CCAs (*n* = 7) and consumers (*n* = 28).

**Results:**

Medicines support visits by CCAs increased from 43/16,863 (0.25 %) to 714/21,552 (3.3 %) (*p* < 0.001). Nurses reported mostly positive experiences, and high levels of trust and confidence in CCAs. They reported that delegating to CCAs sometimes eliminated the need for duplicate nurse and CCA visits (for people requiring personal care plus medicines support) and enabled them to visit people with more complex needs. CCAs enjoyed their expanded role and were accepted by clients and/or carers. Nurses and CCAs reported effective communication when medicine-related problems occurred. No medication incidents involving CCAs were reported. Barriers to implementation included the limited number of CCAs employed in the organisation and reluctance from some nurses to delegate medicines support to CCAs. Enablers included training and support, existing relationships between CCAs and nurses, and positive staff attitudes.

**Conclusions:**

Appropriately trained and supervised support workers can be used to support community nurses with providing medicines management for older people in the home care setting, particularly for those who are at low risk of adverse medication events or errors. The model was acceptable to nurses, clients and carers, and may offer a sustainable and safe and effective future workforce solution to provision of medicines support for older people in the home care setting.

## Background

Increasing numbers of older people are requiring support from community nursing services with managing medicines at home, as a result of population ageing, increasing intensity of medical therapy for chronic diseases (leading to increasing polypharmacy), earlier discharge from hospital, and a shift towards home-based care in place of residential care [1–3].

Reliance on nurses to provide this support may not be sustainable, due to projected workforce shortages and healthcare budget limitations [4, 5]. There is evidence that some medicines support tasks, such as prompting or assisting people to take their medicines, can be undertaken by trained support workers (known by various titles, such as medication aides, personal care workers, nursing assistants) [6–8], and that nurses can successfully delegate medicine support tasks [2, 9–15]. Using support workers to assist with medicine administration within residential care facilities/nursing homes is common in some countries including Australia [6, 9, 10, 14, 16–23]. However, this workforce group is under-utilised in the home care setting [6, 21, 24, 25], and there is little evidence related to their use [3, 21, 26, 27]. Few studies have explored community nurses’ perceptions and experiences with delegating medicine support tasks to support workers [3, 10], or community support workers’ perceptions and experiences with delivering these services.

A recent study in the United Kingdom explored the roles of nursing assistants within community nursing services [27]. It was reported that a lack of consensus regarding the role of nursing assistants had led to wide variation in nursing assistants’ medicines support roles between service providers. However, managers reported that nursing assistants improved workforce flexibility and helped community nursing service providers respond to changing demands on their services. There has been no study of support workers’ medicines support roles in within a community nursing service in Australia.

As part of an Australian government-sponsored aged care workforce reform project, we implemented and evaluated a medicines support role using support workers – referred to in this paper as community care aides (CCAs) - within a large community nursing service. Traditionally most medicine support tasks within the organisation were performed by registered nurses (RNs) and enrolled nurses (ENs), who made up 82 % and 12 % of the workforce respectively. CCAs’ (6 % of the workforce) mainly delivered personal care, and had a limited role in medicines support. The new workforce model, titled ‘WISE Medicines Care’ (Workforce Innovation for Safe and Effective Medicines Care) was designed to enable nurses to delegate medicines support tasks to CCAs.

The primary aims of this study were to assess whether the WISE Medicines Care model could increase the number of home visits for medicines support that were conducted by CCAs, explore nurses’, CCAs’, older peoples’ and carers’ experiences and satisfaction with the CCAs’ expanded role, and identify enablers and barriers to delegation of medicines support to CCAs.

## Methods

### Design

Prospective before-after, mixed-methods (quantitative and qualitative) study.

### Setting

Two metropolitan sites within a large non-profit community nursing service in Victoria, Australia between July 2012 and December 2013.

### Participants

#### Quantitative study

- All home-dwelling older people (50 years or over) who received home visits for medicines support (referred to as clients),
- All nurses who were involved in delivery, delegation and supervision of medicines support services.
- All CCAs (Note: The new workforce model was implemented using the existing four CCAs at each study site; no additional CCAs were employed).

#### Qualitative study

A purposive sample of:

- clients and/or their carers who received medicines support;
- nurses involved in the delivery, delegation and supervision of medicine support; and
- CCAs.

### Intervention

The WISE Medicines Care model was developed by the research team, with input from a broad range of stakeholders, including community nursing and aged care providers, consumers, health professionals, government and professional peak bodies. It introduced person-centred medicines management assessment tools to assist nurses to assess clients’ risk of adverse medication events or errors and determine the level of support required, taking into consideration the client’s goals, health status, types of medicines, physical and cognitive capabilities and availability of informal support. Clients identified as low risk, but still needing assistance, were able to have medicine support visits by a CCA, under (indirect) supervision of a nurse.

The community nursing service’s organisational policies and procedures were revised to enable CCAs to work to an expanded scope of practice in relation to medicines support. This included (when authorised by the primary nurse and documented in the client’s care plan) prompting clients to self-administer medicines, removing medicines from packaging, crushing tablets, and assisting with administration of oral and topical medicines. Practice guidelines were developed to assist CCAs with problem solving potential scenarios that might occur in the home.

Prior to implementation of the model, all CCAs (*n* = 8) undertook competency based training in medicines support that included two Australian Government Industry Skills Council units [28, 29]. Training was delivered face-to-face by an educator who was independent of the research team. CCAs also received workplace training and assessment with an RN. RNs (*n* = 132) and ENs (*n* = 10) participating in the new workforce model were provided with training including a self-directed learning module and a two-hour face-to-face session conducted by the research team. The training introduced the new workforce model and covered assessment of medicines management and delegation and supervision of medicines support tasks to CCAs. Ongoing support for nurses and CCAs in the new model was facilitated by senior clinical staff and the research team. The new workforce model was implemented from February 2013.

### Data collection and analysis

#### Quantitative study

At each home visit, nurses and CCAs electronically recorded care activities undertaken. At the end of the pre- and post-implementation data collection periods (13 weeks each: July-October 2012 and July-October 2013), reports summarising all home visits with one or more medication-related activity code were generated. Data included the type of worker who undertook the visit and activities undertaken. Reports summarising medication incidents (e.g. administration errors) were generated from the organisation’s incident reporting system.

Data analysis was performed using the Statistical Package for Social Sciences (SPSS), Version 21. Data were reported as frequencies and proportions for categorical variables, and median and inter-quartile range [IQR] for discrete variables and continuous variables with skewed distribution. Univariate analyses were used to compare changes/differences between pre- and post-implementation data. Chi-square test was used for categorical variables and Mann–Whitney test for non-normally distributed continuous variables. A p-value of <0.05 indicates statistical significance.

Clients were classified into those receiving longer-term medicines support (>30 days) versus those receiving shorter-term support (≤30 days) and those with less complex care needs versus more complex care needs, for subgroup analyses. ‘More complex care needs’ was defined as: “more than 50 % of visits that included medicines-related activities also involved tasks that would usually be unsuitable for a CCA (e.g. injections, wound care, catheter care or palliative care).”

#### Qualitative study

Twenty-five in-depth interviews were carried out with clients and/or carers during the pre- (*n* = 10) and post-implementation periods (*n* = 15). A mix of clients (English and non-English speaking, more complex and less complex care needs, received medicines support from CCAs [at least 10 visits] and received support from nurses only) were selected. Clients/carers were invited by telephone to participate. Those who agreed were sent a Plain Language Statement and Consent Form, and an interview was arranged. Interviews were conducted face-to-face at the person’s home, with the exception of one which was conducted by telephone. Interviews were conducted using a semi-structured interview guide, and lasted 30–80 mins. For non-English speakers a professional interpreter was used for consent and the interview.

Six focus groups (5–10 participants in each) and an in-depth interview (*n* = 1) were undertaken with staff following the pre- and post-implementation periods (3 focus groups at each time-point). A range of RNs, ENs and CCAs were invited, to represent the staff mix at the study sites. Focus groups were facilitated using a semi-structured interview guide and lasted 50–75 mins. The interview lasted approximately 20 mins.

Participants provided written consent prior to the interviews or focus groups. Clients and/or carers who were unable to provide written consent instead gave audio-recorded verbal consent.

Interviews and focus groups were audio-recorded and professionally transcribed. Transcripts were read and checked against the original recordings to ensure accuracy, then imported into the qualitative data management program QSR NVivo 10/11 for analysis.

Initial coding of transcript data consisted of descriptive (participant group, setting/context) and topic (specific questions/topics) coding. Coding reports were then read by the research team using a constant comparative approach to identify themes. Emergent themes were discussed and interpretations compared to identify key issues. This process was used to enhance accuracy and validity of data interpretation [28].

### Study endpoints

#### Quantitative study

*Primary endpoints:*

- Proportion of home visits for medicines support that were undertaken by a CCA;
- Proportion of clients who received one or more visits by a CCA for medicines support;
- Proportion of home visits for medicines support that were undertaken by RNs and ENs.

*Secondary endpoints:*

- Number of medication-related incidents reported;
- Number of medicine support visits that were undertaken by CCAs for clients with and without more complex care needs;
- Number of medicine support visits that were undertaken by CCAs for short-stay versus longer-stay clients.

#### Qualitative study

- Nurses’, CCAs’ and clients’/carers’ experiences and satisfaction with the new workforce model, and barriers and enablers for CCA role expansion.

### Ethics approval

The study was approved by the Human Research Ethics Committees of Austin Health, Royal District Nursing Service (RDNS) and Monash University.

## Results

### Quantitative study

During the pre-implementation period, 467 older people received medicines support; during the post-implementation period there were 572 people (Table 1).

**Table 1:** Characteristics of older people receiving community nursing service for medicines support

Characteristics of older people	Pre-implementation	Post-implementation	*p*-value
	(*n* = 467)	(*n* = 572)	
Age (years), median (IQR)^a^	82.0 (75.0 − 87.0)	81.0 (73.0 − 87.0)	0.11
Gender (female), n (%)	293 (62.6)	367 (64.2)	0.64
Length of stay > 30 days, n (%)	345 (75.2)	416 (70.5)	0.12
Less complex care needs, n (%)	243 (52)	266 (46.5)	0.08

^a^IQR = interquartile range

#### Primary endpoint

Home visits for medicines support undertaken by CCAs increased significantly from 43/16,863 (0.25 %) to 714/21,552 (3.3 %) - an absolute increase of 3.1 % (95 % CI 2.8-3.3 %; *p* < 0.001) between the pre- and post-implementation periods (Table 2). The increase was greater for study site 1 − increased from 3/7,727 (0.04 %) visits to 538/10,179 (5.3 %) visits (absolute increase 5.3 %; 95 % CI 4.8 − 5.7 %; *p* < 0.001) than study site 2 − increased from 40/9,136 (0.44 %) visits to 176/11,373 (1.55 %) visits (absolute increase 1.11 %; 95 % CI 0.84 − 1.38 %; *p* < 0.001).

**Table 2:** Types of personnel visiting older people at home for medicines support

Medicine support home visits undertaken by	Pre-implementation	Post-implementation	Changes in % of visits (95 % CI)^a^	*p*-value
	(*n* = 16,863 visits)	(*n* = 21,552 visits)		
Registered Nurses, n (%)	15,126 (89.7)	18,733 (86.9)	−2.8 (2.15 − 3.45)	<0.001
Enrolled Nurses, n (%)	1,694 (10.0)	2,105 (9.8)	−0.2 (−0.41 − 0.81)	0.53
Community Care Aides, n (%)	43 (0.25)	714 (3.3)	+3.3 (1.66 − 4.44)	<0.001

^a^The sign indicates the direction of changes i.e. + indicates increase in the proportion of post-implementation visits and – indicates decrease in the proportion of post-implementation visits

CCAs assisted with delivering medicines support for 5 (1.1 %) clients in the pre-implementation period and 27 (4.7 %) clients in the post-implementation period, an absolute increase of 3.6 % (95 % CI 1.43 − 5.77 %; *p* < 0.002).

The proportion of medicine support visits undertaken by RNs decreased by 2.8 % (95 % CI 2.2 − 3.5 %; *p* < 0.001), and there was no change in the proportion of medicine support visits undertaken by ENs (Table 2).

#### Secondary endpoints

There were 29 medication-related incidents reported in the pre-implementation period, and 34 in the post-implementation period. No medication-related incidents involved CCAs.

Subgroup analyses showed that most CCA medicine support visits were for clients with less complex medicine support needs and receiving longer-term care (Table 3).

**Table 3:** Types of clients receiving CCA medicine support home visits

Type of clients	Number of CCA medicine support visits	
	Pre-implementation	Post-implementation
*Complexity of care*		
Required less complex care needs, n	42	711
Required more complex care needs, n	1	3
Total	43	714
*Duration of care*		
Required longer-term care (>30 days)	43	714
Required shorter-term care (≤30 days)	0	0
Total	43	714

### Qualitative study

Twenty-seven nurses (22 RNs, 5 ENs), seven CCAs, 18 older people and 10 carers participated in at least one focus group/interview (Table 4). Six RNs, two ENs and three CCAs participated in both pre- and post-implementation focus groups/interviews. The CCAs had worked at the community nursing service for a median of 18 years (range 2–23 years). Clients were aged 86–98 years; 3 were non-English speaking clients (2 Greek and 1 Arabic speaking); and most (13/18) received regular medicines support visits (≥3 visits per week) from the community nursing service for more than 30 days.

**Table 4:** Participants in focus groups/interviews

Participants	Pre-implementation			Post-implementation		
	No. participants	Gender (F vs M)^a^	Study site (A vs B)^b^	No. participants	Gender (F vs M)^a^	Study site (A vs B)^b^
*Nurses’ focus groups/interview*						
RNs	14	12 F, 2 M	6 A, 8 B	14	10 F, 4 M	6 A, 7 B
ENs	3	3 F	1 A, 2 B	4	4 F	1 A, 3 B
*CCAs’ focus groups*						
CCAs	5	5 F	1 A, 4 B	5	5 F	3 A, 2 B
*Older people’s and/or carers’ interviews*						
Older people	10	4 F, 6 M	8 A, 2 B	8	7 F, 1 M	5 A, 3 B
Carers of participating older people	6	5 F, 1 M	5 A, 1 B	4	2 F	3 A, 1B

^a^F = Female; M = Male

^b^A = Study site 1; B = Study site 2

### Perceptions and satisfaction with CCAs’ medicine support role

#### Nurses

Prior to the new workforce model, nurses reported a range of views about delegating medicines support tasks to CCAs. These views acknowledged the high cost associated with the current RN model of care and concern about whether CCAs would have the ability to recognise problems that the client may be experiencing.

“The CCAs are competent… especially if the risk is low. It’s very expensive having RNs go in to give people Panadol [paracetamol] and that sort of stuff.” (RN 2)

“It might increase the workload of the RN if they [CCAs] go in and discover little problems.... because you’ll [need to] be troubleshooting.” (RN 3)

After implementation of the new workforce model, nurses reported mostly positive experiences. They reported high levels of trust and confidence in CCAs, effective communication between nurses and CCAs, and expressed a desire to have more CCAs.

“I visited someone today that the CCA’s been seeing regularly and it’s not a problem, and I have to say the documentation from the CCA is brilliant, they’re terrific, and they always ring if there’s an issue.” (EN 3)

“…..just wish we had more of them.” (RN 23)

Nurses noted that using CCAs sometimes eliminated the need for duplicate visits for people who required medicated creams.

“I found it really helpful because often they’re making hygiene visits, and every now and then someone will break out in a rash and need a cream, and in the past they come to do the shower and then I have to go and do the cream, … and so that’s a really good opportunity for them to be able to go…. I think that’s been really positive.” (RN 23)

Nurses noted that CCAs enjoyed their new role, and were accepted by older people/carers

“I think it’s been good to educate the clients as well that they don’t maybe need a nurse, that there is someone in that caring role that can still do the same thing……” (RN 9)

Some nurses reported being freed up to spend more time with, or to see more, older people with complex needs:

“I’ve got a new PD [peritoneal dialysis] client in the morning, I have to be at his [home] ..... between 8 am and 9 am but then I also have medication that I need to do before 10 am or 11 am and it’s just never going to happen. So it’s good to know that I can reallocate them to the CCAs.” (RN 16)

### CCAs

Prior to the new workforce model, CCAs were receptive of the idea of doing more medicines support work and were keen to learn new skills.

“I think it’s good to have/stretch as another skill.” (CCA 2)

After implementation of the new workforce model, CCAs reported mostly positive experiences with providing medicines support and the changes to their role.

“…..I love it.” (CCA 1)

“I think I could do more.” (CCA 4)

CCAs reported that their expanded role was well accepted by most nurses and that they had effective communication with the nurses and were able to report problems to them.

“if you’re at a client’s house and you’ve got a problem with that med[icine] then you’d either ring the primary nurse for that client or just ring the centre.” (CCA 6)

“…..one day I went in [to a client’s home] I picked up the DAA [dose administration aid] and all these pills went all over the table because it was already opened, so I had to call the RN to come out and sort it out.” (CCA 1)

### Clients/carers

Prior to the new workforce model, clients and/or their carers expressed a range of views when asked about their perception of CCAs assisting them with their medicines. The majority expressed positive or neutral views, where they indicated that they would accept the CCAs if the CCAs have received appropriate training, or that it would make no difference what type of workers visited them.

‘It wouldn’t worry me. As long as I take my medication properly I don’t care who it was.’ (Client 11)

One carer noted potential benefits of utilising CCAs for less complex medicine support tasks, as this would free up nurses and enable the nurses to undertake more complex medicines tasks.

“I think it’s a great idea…for mum she doesn’t need a nurse to come here to give her tablets, as long as someone gives them, they don’t have to be as highly skilled as a nurse, wasting nurses’ time to come and give tablets…..and there’s so many other patients out there that probably need the nurse more than mum at this stage…” (Carer 3)

After implementation of the new workforce model, clients/carers who had received medicines support from CCAs were sometimes unable to identify the type of workers assisting them, but reported satisfaction with the support received.

“I don’t know their names…I sort of expect them and I just let them in…They’re very nice, very good, pleasant…they only come to make sure I have my tablets…. it doesn’t make any difference to me [whether it is a CCA or a nurse]…I’m very happy with them…” (Client 20)

“They had a nurse coming on the Monday, to check that week’s pills were correct…And then the other days of the week it was a care aide …Different people, it didn’t seem to be a problem…there’s no difference - not just for dishing out the tablets anyway…” (Carer 17)

Some clients indicated that they were comfortable with the idea that nurses should see people who were sicker or required more complex care.

“I feel comfortable…that was the whole idea that I would get the CCA and the nurses go and do more difficult jobs like changing people’s dressings or stitches or some sort of real nursing jobs…” (Client 14)

Only one carer expressed concerns about utilising CCAs to assist with medicines support. This was mainly due to concerns about the type of training provided to CCAs.

“… it would depend on how good the training that’s given… It’s a big thing, it’s not just Hi, I am here to give your tablets….” (Carer 21)

#### Barriers and enablers to implementation of the new workforce model

A range of factors affecting the use of CCAs for medicines support were identified prior to and after implementation of the new workforce model.

### Barriers

#### Limited number of CCAs

The limited number of CCAs employed in the organisation, together with large distances travelled in some areas, were perceived by nurses to be barriers to using CCAs for medicines support.

“I have got a number of clients that probably would be suitable, but I don’t have a CCA in my area.” (EN 2)

“…the travel distance…you can’t just allocate them one down, you’ve got to really look at who’s in the area and not have too much travel…” (RN 18)

#### Nurses’ attitudes

CCAs reported that some nurses were reluctant to delegate medicines support visits to them as nurses didn’t like the perceived loss of control in doing medicines support visits. Nurses reported that they were concerned about the increased risk associated with delegating medicines support to a CCA (e.g. concerns that CCAs may not identify client deterioration), however no examples of such situations were reported.

“it’s frustrating…I have scope but they’re not giving me medications…say with the creams… for whatever reason they’re not handing it over so it’s just making a job doubly hard… I find it completely pointless that I just go and wash off cream that they’ve put on or that the nurses are actually doing a double visit to come back after me….”’ (CCA 5)

“I think initially there was a lot of negativity within the field staff [nurses], because some people felt that they [CCAs] were actually taking a Division 1 or Division 2’s [RN’s and EN’s] role, but without the education, but after actually showing them [nurses] the work book [client visit record], they felt comfortable, and it was a matter of educating the field staff [nurses] that they [CCAs] were capable of actually administering from a Webster pack [dose administration aid].” (RN 6)

“I don’t like doing it [delegating to a CCA] sometimes when you see a large number of meds in the Webster Pack [dose administration aid], you think I’m not sure if this is right for CCAs to be prompting so many medications.” (RN 15)

### Enablers

#### Time and support for CCAs

Prior to implementation of the new model, CCAs felt they would require time, and support from the nurses, to develop confidence in doing medicines support visits.

“I think having the time to go out with the RNs and do some medications with them, and be able to sit down with them and go through and make sure…I think once we do the practice, we’ll become good at it…” (CCA 2)

“…. we will need time, at least initially, for us to be able to feel [confident].” (CCA 4)

#### Experienced CCAs

CCAs who participated in the new workforce model were already colleagues of the nurses, which facilitated successful implementation of the expanded CCA role.

“We’ve had CCAs for a long time and …. we’ve always had a good communication system with them and they know their roles and they know their limitations and they’re very quick to call for help.” (RN 18)

#### CCAs’ positive attitudes

Nurses viewed that the CCAs were very positive in accepting the new role of undertaking medicines support visits.

“I think their confidence with the education that they received, and they were so eager to put it into place, and being given that extra responsibility.” (RN 9)

“They seem positive about it…they want something different to doing showers all the time.” (RN 23)

#### Nurses’ positive attitudes

CCAs noted that nurses became more supportive of their expanded role once they saw what the CCAs were doing, and that when nurses were supportive of the CCAs’ role they were allocated more medicines support visits.

“We had one or two care managers that were really enthusiastic and others weren’t….but I think now they see that it’s working….we’ve got a lot [of clients] on now.” (CCA 6)

#### Educating older people/carers

Some nurses suggested that educating clients and carers about the extended CCA role helped improve acceptance of CCAs.

“I think it’s been good to educate the clients as well that they don’t maybe need a nurse, that there is someone in that caring role that can still do the same thing…for people to realise they don’t need a nurse to have to be there to be able to do something like tablets.” (RN 9)

#### Presence of an advocate or champion

At one site (the site that had a smaller increase in CCA medicines support visits – study site 2) it was noted that there was no advocate or ‘champion’ for the CCAs, to support and drive practice change. At the site that had greater success in shifting medicines support tasks to CCAs (study site 1) there was a senior nurse who met regularly with the CCAs and allocated clients to them for medicines support.

“He [X] is not just our medications supervisor, he’s a grade 4 that’s in charge of us so we have CCA meetings where we get together with him we might go out for lunch or coffee or have a meeting at the centre.” (CCA 6)

“…You have a person [X] [at your site]. I don’t have a person that I can go to.” (CCA 5)

“I [X] help support the CCAs, we have meetings every second week and they seem to be, they’re happier about their role and happier about their job doing the medications, it’s actually enhanced them and they’ve got more knowledge, and it can actually be taken to any other facility they need.” [RN 6]

## Discussion

The WISE Medicines Care workforce model enabled nurses to delegate medicine support tasks to CCAs working in an expanded role within a community nursing service. The success of the model is illustrated by the evidence that, without increasing the number of CCAs employed at the study sites, the number of medicines support visits undertaken by CCAs increased significantly. One-third of CCA medicine support visits were combined with other care (e.g. personal care), avoiding the need for an extra visit by a nurse. The other two-thirds were for medicines support only. These extra visits were possible because CCAs had previously been underutilised and the medicines visits were for clients who resided in the local area of the CCAs’ other clients, minimising additional travel time.

Subgroup analyses indicated that CCAs mainly visited older people who were receiving long-term medicines support and whose care needs were less complex. These people would be expected to be at relatively low risk of adverse medicines events compared with more complex and short-term clients (e.g. post-acute care, palliative care and diabetes management) who continued to be managed by nurses. Delegation of medicine support tasks for low risk clients to CCAs allowed nurses to focus on older people with more complex needs. No medication incidents were reported involving CCAs, suggesting that the model did not adversely impact on patient/client safety.

Prior to implementation, nurses and CCAs indicated that they were willing to trial the new workforce model but expressed some concerns about the extended CCA role. Clients/carers had mixed perceptions regarding CCA roles. After implementation of the new model all groups reported mostly positive experiences. CCAs enjoyed working in the model and most nurses felt comfortable with delegating to and supervising CCAs. The WISE Medicines Care implementation and training enhanced nurses’ understanding and acceptance of CCAs’ medicines support role. Clients/carers agreed that CCAs could be utilised for less complex medicines support tasks.

CCAs’ medicines support activities in this study included reminding, prompting and assisting with administration of medicines supplied in pharmacy-packed dose administration aids (DAAs) and some medicated creams, patches, eye drops and inhalers. Current Australian guidelines and policies are more restrictive with respect to what medicines support tasks nurses can delegate to unregulated health workers such as CCAs, limiting support workers to assisting clients with self-administration of medicines from a pharmacy packed DAA [29, 30]. This restriction precludes using CCA’s to support older people with low risk, non-complex medicines regimens supplied in original packaging (including topical medicines, transdermal patches and simple oral medication regimens), unnecessarily increasing the cost of care and medicines supply to the person.

A recent Australian study explored the perspectives of nurses and personal carers (PCs) from residential and community-based aged care services about PCs’ role in medications [21]. The study reported that nurses had mixed perceptions about PCs’ role in medications. While PC participants (*n* = 3) were limited to those working in residential care, the study included community nurses’ perspectives on PCs’ medication roles in the community setting. Nurses felt that community PCs could only assist with administering medications packed into dose administration aids (DAAs) by a pharmacy, and any assistance beyond this would require the client’s medical practitioner’s authority. Conversely, the study identified that residential care PCs had a greater role in medications (e.g. checking patients’ medications, crushing and mixing medications, assisting with administration of topical products and inhalers). The need for upskilling PCs to assist with medicine support (due to budget limitations and workload pressure) was highlighted [21]. Another study reported that residential care PCs undertook more complex medicine tasks (e.g. assessing and making decisions about administering analgesia, monitoring pain) [19]. Our study provides evidence that the community PCs (CCAs) could undertake medicines support tasks, under the direction of nurses, similar to those undertaken by residential aged care PCs.

A few studies have examined care workers’ involvement in medicines support in the community [3, 26, 27]. None were conducted in Australia, so their findings may not be generalisable to the Australian community care setting. In Sweden, a survey of Home Care Aides (HCAs) identified that most were engaged in medicine administration [26]. Another Swedish study explored the perception of community nurses towards delegating medication administration to HCAs, and identified several factors influencing nurses’ decisions [3]. Because they had a large workload and were working with many HCAs, nurses viewed their role in delegating medication support to HCAs as important. Barriers to delegating medicine administration to HCAs included: lack of clear guidelines/decision frameworks for nurses’ delegation, and poor understanding among organisation authorities about nurses’ role and responsibility in delegation. In our study, nurses expressed similar concerns before they undertook education and training on the new model and their roles and responsibilities in delegation and supervision, however training and organisational support helped to address these concerns. In the Swedish study, effective communication was noted as an important factor that enabled nurses to establish trust and confidence in the HCAs, [3] which is consistent with our study.

A study in the United Kingdom explored the roles of nursing assistants within 102 providers of community nursing services and identified a range of tasks undertaken by the nursing assistants including medicines support tasks [27]. However, they reported wide variation in practice among the service providers, in which some nursing assistants were involved in the provision of more complex medicine support/nursing tasks under nurses’ supervision (e.g. administering insulin to stable patients, changing fentanyl patches, conducting reassessments), and some provided only basic medicine support tasks (e.g. reminding patients to take medications).

Several studies investigated associations between care workers’ involvement in medicine support and a range of outcomes in residential care and hospital settings [14, 31–35]. They reported evidence that care workers’ involvement in medicine support could lead to improved job satisfaction for both nurses and care workers, as well as improved people’s satisfaction with care, and improved quality of care by reducing workload pressure on nurses, enabled nurses to spend more time undertaking complex nursing activities [14, 20, 31, 33, 34]. Similarly, our study found that an expanded CCA role in medicines support resulted in improved job satisfaction for CCAs, that nurses and clients were generally comfortable with the role, and that it sometimes freed up nurses to spend time on more complex nursing tasks.

Our study found that delegating medicines support to CCAs did not increase the rate of medication incidents. Several studies have reported similar findings [31, 34, 35], while others have reported medication incidents associated with use of care workers [9, 20, 36, 37] or have been inconclusive [8, 14, 15]. Consistent with our findings, previous studies have reported that using care workers for medicines support could provide opportunities for cost-saving [8, 31, 32].

Our study had several strengths and limitations. Strengths included using a mixed-methods approach to provide a broad and detailed understanding of the model, and including two study sites with different workforce mix and client populations to enhance generalisability. Although the workforce model was tested within a single community nursing organisation, which may limit generalisability of our findings, we included a broad range of external stakeholders in the development of the workforce model to enhance its transferability and sustainability.

Most CCAs had been working for the organisation for several years and had established relationships with the nurses. This facilitated implementation of the new model, but may limit generalisability of our findings to situations where less experienced CCAs are employed for the role. Another limitation is that medication safety outcomes were only assessed by analysing incident reports, which rely on staff identifying and reporting incidents, potentially resulting in under-estimation of error rates. However, this was the case in both the pre- and post-implementation periods. The quantitative evaluation utilised data that was recorded by nurses and CCAs, and were not independently verified. Recording of medicines support visits may have improved in the post-implementation period as a result of education received by nurses and CCAs, so some of the increase in medicines support visits may be a result of better recording. We recruited a diverse range of clients (in terms of language spoken, type of medicines support), but there may have been some selection bias in our recruitment for client/carer interviews.

## Conclusion

Appropriately trained and supervised support workers can be used to support community nurses with providing medicines management for older people in the home care setting, particularly for people who are at low risk of adverse medication events or errors. The model was acceptable to nurses, clients and carers, and may enable nurses to work to the top of their scope of practice, and may offer a potentially sustainable and safe and effective future workforce solution to provision of community home care for an ageing population.

## Abbreviations

RNs: Registered nurses
ENs: Enrolled nurses
CCAs: Community care aides
HCAs: Home care aides
PCs: Personal carers
RDNS: Royal district nursing service
WISE Medicine Care: Workforce innovation for safe and effective medicine care
GP: General practitioner
DAAs: Dose administration aids

## References

[1] Nay R, Garratt S, Koch S (1999). Challenges for Australian nursing in the international year of older persons. Geriatr Nurs.

[2] Johansen E, Fagerstrom L (2010). An investigation of the role nurses play in Norwegian home care. Br J Community Nurs.

[3] Craftman AG, von Strauss E, Rudberg SL, Westerbotn M (2013). District nurses’ perceptions of the concept of delegating administration of medication to home care aides working in the municipality: a discrepancy between legal regulations and practice. J Clin Nurs.

[4] Cowin L, Jacobsson D (2003). Addressing Australia’s nursing shortage: is the gap widening between workforce recommendations and the workplace?. Collegian.

[5] Health Workforce Australia (HWA) (2012). Health Workforce 2025 – Doctors, Nurses and Midwives.

[6] Budden JS (2012). A national survey of medication aides: education, supervision, and work role by work setting. Geriatr Nurs.

[7] Health Workforce Australia (HWA) (2012). Workforce innovation: caring for older people program - final report.

[8] Kessler I, Spilsbury K, Heron P. Developing a high-performance support workforce in acute care: innovation, evaluation and engagement. Health Serv Deliv Res 2014;2(25). http://www.journalslibrary.nihr.ac.uk/__data/assets/pdf_file/0013/124060/FullReporthsdr02250.pdf. 25642534

[9] Dickens G, Stubbs J, Haw C (2008). Delegation of medication administration: an exploratory study. Nurs Stand.

[10] Reinhard SC, Young HM, Kane RA, Quinn WV (2006). Nurse delegation of medication administration for older adults in assisted living. Nurs Outlook.

[11] Culver J (2007). Medications guidelines clarify RN role in aged care. Lamp.

[12] Mitty E, Flores S (2007). Assisted living nursing practice: medication management: part 2 supervision and monitoring of medication administration by unlicensed assistive personnel. Geriatr Nurs.

[13] Mitty E, Resnick B, Allen J, Bakerjian D, Hertz J, Gardner W (2010). Nursing delegation and medication administration in assisted living. Nurs Adm Q.

[14] Randolph PK, Scott-Cawiezell J (2010). Developing a statewide medication technician pilot program in nursing homes. J Gerontol Nurs.

[15] Spellbring AM, Ryan JW (2003). Medication administration by unlicensed caregivers: a model program. J Gerontol Nurs.

[16] Annoymous: Personal care workers to administer drugs. Australian Nursing Journal 2006, 14:6. 16848248

[17] Carder PC (2012). “Learning about your residents”: how assisted living residence medication aides decide to administer pro re nata medications to persons with dementia. Gerontologist.

[18] Glazer G (2002). Medication administration interventions that must be performed by a registered nurse. Online J Issues Nurs.

[19] Holloway K, McConigley R (2009). Descriptive, exploratory study of the role of nursing assistants in Australian residential aged care facilities: the example of pain management. Australas J Ageing.

[20] Hughes CM, Wright RM, Lapane KL (2006). Use of medication technicians in US nursing homes: part of the problem or part of the solution?. J Am Med Dir Assoc.

[21] Tan A, Emmerton L, Hattingh L (2012). Personal carers’ role in medication administration: An exploratory study in a rural community in Queensland. Aust Nurs J.

[22] Mitty E (2009). Medication management in assisted living: a national survey of policies and practices. J Am Med Dir Assoc.

[23] Lee CY, George J, Elliott RA, Chapman CB, Stewart K (2011). Exploring medication risk among older residents in supported residential services: a cross-sectional study. J Pharm Pract Res.

[24] Bradford J (2012). Medication administration in the domiciliary care setting: whose role?. Br J Community Nurs.

[25] Australian Nursing Federation (ANF) (2009). Balancing risk and safety for our community: unlicensed health workers in the health and aged care systems.

[26] Axelsson J, Elmstahl S (2004). Home care aides in the administration of medication. International J Qual Health Care.

[27] Spilsbury K, Pender S, Bloor K, Borthwick R, Atkin K, McCaughan D, Watt I, Adderley U, Wakefield A, McKenna H: Support matters: a mixed methods scoping study on the use of assistant staff in the delivery of community nursing services in England. *Health Serv Deliv Res* 2013;1(3). http://www.journalslibrary.nihr.ac.uk/__data/assets/pdf_file/0003/70869/FullReport-hsdr01030.pdf. 25642505

[28] Pope C, Ziebland S, Mays N (2000). Qualitative research in health care. Analysing qualitative data. BMJ.

[29] Australian Nursing and Midwifery Federation (ANMF) (2013). Nursing Guidelines: Management of Medicines in Aged Care.

[30] Australian Government Department of Veterans’ Affairs (DVA) (2010). Guidelines for the provision of community nursing services.

[31] Lee TY, Yeh ML, Chen HH, Lien GH (2005). The skill mix practice model for nursing: measuring outcome. J Adv Nurs.

[32] Burruss RA, Ashworth DN, Arikian VL (1993). Medication administration by non-RN personnel: a safe and cost-effective response to the RN shortage. Health Care Supervisor.

[33] Walker MJ (2008). Effects of the medication nursing assistant role on nurse job satisfaction and stress in long-term care. Nurs Adm Q.

[34] Walsh JE, Lane SJ, Troyer JL: Impact of medication aide use on skilled nursing facility quality. *The Gerontologist* 2013: Epub ahead of print [doi:10.1093/geront/gnt1085]. 10.1093/geront/gnt085 23969257

[35] Scott-Cawiezell J, Pepper GA, Madsen RW, Petroski G, Vogelsmeier A, Zellmer D (2007). Nursing home error and level of staff credentials. Clin Nurs Res.

[36] Young HM, Gray SL, McCormick WC, Sikma SK, Reinhard S, Johnson Trippett L (2008). Types, prevalence, and potential clinical significance of medication administration errors in assisted living. J Am Geriatr Soc.

[37] Kapborg I, Svensson H (1999). The nurse’s role in drug handling within municipal health and medical care. J Adv Nurs.

